# VarR controls colonization and virulence in the marine macroalgal pathogen *Nautella italica* R11

**DOI:** 10.3389/fmicb.2015.01130

**Published:** 2015-10-13

**Authors:** Melissa Gardiner, Neil D. Fernandes, Dennis Nowakowski, Mark Raftery, Staffan Kjelleberg, Ling Zhong, Torsten Thomas, Suhelen Egan

**Affiliations:** ^1^School of Biotechnology and Biomolecular Sciences, Centre for Marine Bio-Innovation, The University of New South WalesSydney, NSW, Australia; ^2^Bioanalytical Mass Spectrometry Facility, Mark Wainwright Analytical Centre, The University of New South WalesSydney, NSW, Australia; ^3^Singapore Centre on Environmental Life Sciences Engineering, Nanyang Technological University, SingaporeSingapore

**Keywords:** bacterial biofilms, *Delisea pulchra*, seaweed disease, macroalgae, microbial interactions, quorum sensing, solo LuxR-type regulator

## Abstract

There is increasing evidence to suggest that macroalgae (seaweeds) are susceptible to infectious disease. However, to date, little is known about the mechanisms that facilitate the colonization and virulence of microbial seaweed pathogens. One well-described example of a seaweed disease is the bleaching of the red alga *Delisea pulchra*, which can be caused by the bacterium *Nautella italica* R11, a member of the Roseobacter clade. This pathogen contains a unique *luxR*-type gene, *varR*, which we hypothesize controls its colonization and virulence. We show here that a *varR* knock-out strain is deficient in its ability to cause disease in *D. pulchra* and is defective in biofilm formation and attachment to a common algal polysaccharide. Moreover complementation of the *varR* gene *in trans* can restore these functions to the wild type levels. Proteomic analysis of bacterial cells in planktonic and biofilm growth highlight the potential importance of nitrogen scavenging, mobilization of energy reserves, and stress resistance in the biofilm lifestyle of *N. italica* R11. Moreover, we show that VarR regulates the expression of a specific subset of biofilm-associated proteins. Taken together these data suggest that VarR controls colonization and persistence of *N. italica* R11 on the surface of a macroalgal host and that it is an important regulator of virulence.

## Introduction

Macroalgae are major habitat formers and contribute to the primary production in temperate marine ecosystems (Jones et al., 1994; Phillips, 2001; Smale et al., 2011). However, there is evidence to suggest that microbial disease is a possible factor contributing to the decline of healthy, macroalgal populations (Correa, 1996; Connell et al., 2008; Wernberg et al., 2009, 2011; Gachon et al., 2010; Campbell et al., 2011). Environmental changes, including increasing seawater temperatures, can reduce innate defense strategies in macroalgal hosts (Potin et al., 2002; Goecke et al., 2010), and evidence also suggests that this results in susceptibility to colonization and infection by pathogens (Harvell et al., 1999; Gachon et al., 2010; Campbell et al., 2011; Case et al., 2011; Koch et al., 2013). Whilst very little is known regarding the specific virulence mechanisms employed by seaweed pathogens, suitable models are being developed to address this issue (Hollants et al., 2013; Egan et al., 2014).

One of the best-studied models for disease in macroalgae is the bacterial-induced bleaching of the red alga *Delisea pulchra* (Campbell et al., 2011; Case et al., 2011; Fernandes et al., 2011; Gardiner et al., 2015), which has significant negative consequences for the health and fecundity of the algal population (Campbell et al., 2014). Two bacterial pathogens, *Nautella italica* R11 (formerly *Ruegeria* sp. R11) and *Phaeobacter* sp. LSS9, have been identified to induce the bleaching disease *in vivo* and *in vitro* (Campbell et al., 2011; Fernandes et al., 2011), and for *N. italica* R11 the infection process was shown to be temperature dependent (Case et al., 2011). Comparative genome analysis of these two pathogens revealed the presence of a gene with homology to LuxR-type transcriptional regulators (termed *varR* here), which was absent from other closely related, non-pathogenic strains (Fernandes et al., 2011). The protein encoded by *N. italica* R11 *varR* (EEB72782) possesses the autoinducer-binding (pfam03472) and transcriptional-activator (pfam00196) domains characteristic of LuxR-type response regulators (Fernandes et al., 2011).

Quorum sensing (QS) systems, including LuxR-type QS, can mediate host colonization and disease induction in several well-studied pathogenic bacteria by coordinating the expression of virulence genes in bacterial populations (Parsek and Greenberg, 2000; Fuqua and Greenberg, 2002; von Bodman et al., 2003; Ham, 2013). The classical LuxR-type QS system involves the LuxI-dependent production of an acylated-homoserine lactone (AHL) signal molecule that binds a response regulator (LuxR) under high cell density and modulates expression of genes under QS control (Fuqua and Greenberg, 2002). However, a subfamily of *luxR*-type genes that are not genetically adjacent to a *luxI* gene, termed solo *luxR*s (Subramoni and Venturi, 2009a) are widespread in Proteobacteria species (Case et al., 2008; Patankar and González, 2009; Subramoni and Venturi, 2009b). Characterized examples of solo LuxR proteins have a range of ligands; from AHLs produced by non-adjacent *luxI* genes (Marketon et al., 2003; Lequette et al., 2006; McIntosh et al., 2008), to host derived, non-AHL small molecules (Zhang et al., 2007; Ferluga and Venturi, 2009; Subramoni et al., 2011; González and Venturi, 2013; Patel et al., 2013). In addition, solo LuxR-type regulators can function in the absence of a signal molecule (Cox et al., 1998). The *N. italica* R11 *varR* gene is not located within an operon and, has the genomic characteristics of a solo *luxR* as it is not adjacent to either of the two *luxI* homologs possessed by this bacterium (Fernandes et al., 2011). The conservation of *varR* across the two characterized macroalgal pathogens is particularly relevant here as the chemical defense molecules (i.e., furanones) produced by *D. pulchra* are AHL-antagonists and bacterial bleaching is environmentally linked to a decrease in algal furanone concentration under increased seawater temperatures (de Nys et al., 1993; Manefield et al., 2002; Campbell et al., 2011).

Colonization of host surfaces facilitates bacterial interactions with macroalgae (Egan et al., 2013), and biofilm formation is a prerequisite in the pathogenesis of *N. italica* R11 (Case et al., 2011). We therefore hypothesized that *varR* may have a key role in the infection and/or colonization of *N. italica* R11. Using a combination of allelic exchange mutagenesis, physiological characterization, and high-throughput proteomics, we show here that VarR regulates several aspects of colonization during *N. italica* R11 pathogenesis. This is the first study to demonstrate a functional role for a luxR-like regulator in a bacterial-induced, macroalgal disease and speaks to the importance of surface colonization by marine bacterial pathogens in the health outcome of macroalgae.

## Materials and methods

### Media and growth conditions used in this study

*Escherichia coli* strains were grown in LB medium supplemented with kanamycin (85 μg ml^−1^), chloramphenicol (34 μg ml^−1^), or gentamicin (50 μg ml^−1^) as appropriate (Table S1). For the *hemA* autotrophic *E. coli* ST18 strains (Table S1) the media was supplemented with aminolevulinic acid (ALA; 50 μg ml^−1^; Thoma and Schobert, 2009). *N. italica* R11 strains were maintained at room temperature in marine broth 2216 (Difco, Becton Dickson USA) with the addition of chloramphenicol (2.5 μg ml^−1^), or gentamycin (50 μg ml^−1^), or both as appropriate (Table S1). The bacterial cultures used for biofilm analysis, attachment assays, and proteomics experiments were grown in bromide-deficient artificial seawater (Br-ASW; Case et al., 2011) supplemented with 10% half strength marine broth (Difco, Becton Dickson USA), termed seawater minimal media (SMM).

### Mutagenesis and complementation of *VarR* in *N. italica* R11

A *N. italica* R11 *varR* allelic replacement mutant strain, termed Δ*varR* here, was constructed by combining the Splicing by Overlap Extension PCR (SOE-PCR) strategy (Horton, 1995) (Table S2) with bi-parental conjugation (Thoma and Schobert, 2009). Experimental details are provided within the Supplementary Materials and Methods; in short, a single homologous recombination event between the SOE-PCR fragment and the genome of the *N. italica* R11 generated the chloramphenicol resistant mutant strain, Δ*varR*. Doubling times for the wild type (WT) and Δ*varR* strains were 128 ± 20 and 124 ± 10 min, respectively, and not statistically different from each other (*p* = 0.46, Students *t*-test).

The Δ*varR* strain was complemented with the WT *varR* gene cloned into the broad-host-range plasmid vector pBBR1 MCS-5 (Kovach et al., 1995) in *E. coli* ST18. Bi-parental conjugation was employed to deliver the WT gene into the Δ*varR* recipient to yield a complemented strain (CΔ*varR*; Supplementary Materials and Methods).

### *D. pulchra In vitro* infection assays

The infection assay of laboratory-cultured algae was performed as outlined by Case et al. (2011) with minor modifications. Briefly, *D. pulchra* spores were grown in Br-ASW for 6 weeks to generate chemically undefended (furanone-deficient) sporelings. Epiphytic bacteria were removed by treating the sporelings with penicillin G (10 μg ml^−1^), streptomycin (10 μg ml^−1^), and kanamycin (20 μg ml^−1^) overnight prior to the assay. The sporelings were then rinsed extensively with Br-ASW to remove antibiotics. The rinsed *D. pulchra* sporelings were then inoculated with 10^6^ CFU ml^−1^ of *N. italica* R11 WT, Δ*varR*, or CΔ*varR* that had been grown for 16 h and rinsed three times with Br-ASW. The assays were performed in triplicate using Costar® six well plates (Corning, USA) and incubated at 24°C with shaking at 25 rpm for 5 days. Characteristic symptoms of biofilm formation, bleaching and invasion were observed with an Olympus BX5OF-3 light microscope (Olympus, Japan). At least five randomly selected fields of view at 45-fold magnification were examined. Disease was defined by the damaged caused to the algal tissue, including fading or bleaching of algal cells, and bacterial invasion associated with a pronounced biofilm. Invasion was defined as the presence of bacteria between and/or within algal cells. Bleaching or fading was defined as localized loss of photosynthetic pigments in algal cells when colonized by bacterial biofilms. Triplicate un-inoculated *D. pulchra* sporelings incubated at 24°C for 5 days were employed in each experiment as controls.

### Attachment to carrageenan

The κ-carrageenan matrix was prepared by dissolving a 5% w/v solution of κ-carrageenan (Sigma-Aldrich) in 50% ASW:50% phosphate buffered saline (PBS) v/v and autoclaving 24 h prior to use. After the solution had cooled to 80°C, 50 μl was added to polystyrene Costar® 96 well plate (Corning) and the gel matrix was solidified at 4°C. The *N. italica* R11 strains were grown to an OD (Abs_600nm_) = 1, washed twice and re-suspended in sterile ASW before 50 μl aliquots were added to respective wells of the κ-carrageenan-coated well plate. The plate was incubated for 6 h at 25°C with gentle shaking (60 rpm) before non-attached cells were removed by gently rinsing the wells six times with sterile PBS. Twenty-five microliters of a 200 μg ml^−1^ solution of trypsin was then added to each well and the plate incubated at 37°C for 5 min to detach cells, which were then counted by dark field microscopy as described previously (Gardiner et al., 2014). Triplicate wells of a κ-carrageenan coated well plate were inoculated with individual *N. italica* R11 strains, and the experiment was replicated three times. Statistical significance was assessed using an ANOVA in GraphPad Prism 4.

### Analysis of biofilm formation

*N. italica* R11 WT, Δ*varR*, and CΔ*varR* were grown in a continuous flow-through biofilm flow cell to allow the development of biofilms on glass slides for analysis by confocal microscopy. Flow cells (three channels each) were prepared according to the method outline in Mai-Prochnow (2007). Silicon tubing was used to connect the flow cell to the media source, with SMM supplied at a continuous flow rate of 0.2 mm s^−1^. Each channel of the flow cell was inoculated with 0.5 ml of cells grown to an OD (Abs_600_) = 0.6 and the flow cells were inverted for 1 h to allow for attachment. The three dimensional structure of biofilms formed at 24, 48, and 72 h post inoculation were visualized using a Fluoview FV1000 Confocal Laser Scanning Microscope (Olympus, USA). The adherent cells were stained with LIVE/DEAD BacLight bacterial viability kit (Molecular Probes, Invitrogen, USA) and dual 488/543 nm filters were used to visualize both live (green) and dead (red) cells using a Z-stack. Five fields of view were captured for each replicate experiment, with a total of three independent experiments conducted for each strain. Images were processed using IMARIS software (Bitplane AG, Switzerland) to calculate the biofilm thickness and biofilm volume for each field of view. Statistical significance was assessed using a univariate permutational MANOVA (with a Euclidean distance resemblance matrix) and pairwise analysis of the interactions in PRIMER 6 (PRIMER-E Ltd).

### Proteomic analysis of *N. italica* R11 WT and **Δ***varR* under planktonic and biofilm growth

The biofilm-associated and planktonic proteome for *N. italica* R11 WT and Δ*varR* strains were profiled using iTRAQ™ (AB SCIEX, USA) labeled quantitative mass spectrometry. Ten milliliter planktonic cultures of the *N. italica* R11 strains were grown for 72 h until they reached stationary phase, and cells harvested by centrifugation and re-suspended in 100 μl ice-cold, molecular-biology grade water (MBW; Eppendorf) by vortexing for 60 s. Cell lysis was confirmed by plating the suspension on half strength MB agar plate and monitoring growth at RT for 3 days. For the biofilm experiments, planktonic cultures were grown as described above, 100 μl was then harvested, re-suspended in fresh media, and used to inoculate 10 cm of Masterflex® platinum-cured silicone tubing L/S® 16 with an inner diameter of 3.2 mm (Cole-Parmer Instrument Co., USA). The tubing was connected to a peristaltic pump (model 323 S; Watson Marlow Bredel pump, England) that was switched on following incubation for 1 h to facilitate attachment of the cells to the tubing. SMM was delivered through the continuous flow-through biofilm system at a flow rate of 0.2 mm s^−1^ for 72 h. To harvest the biofilm cells the tubing was aseptically cut and loosely attached cells were discarded by gently rinsing three times, before the biofilm biomass was lysed as described above for the planktonic cells. One hundred microliters of the crude protein extracts from *N. italica* R11 WT and Δ*varR* cells grown under planktonic or biofilm conditions (referred to herein as WT biofilm, WT planktonic, Δ*varR* biofilm, and Δ*varR* planktonic) were labeled with iTRAQ™ reagents and quantified using mass spectrometry in triplicate independent experiments. The reduction, alkylation and trypsinization of the crude protein extracts, and subsequent peptide labeling with iTRAQ™ reagents (AB SCIEX, Foster City, USA) was performed as described previously (Matallana-Surget et al., 2009; Supplementary Materials and Methods). The labeled peptides were purified using strong cation exchange (SCX) chromatography, desalted, and dissolved in 0.05% HFBA (heptafluorobutyric acid)/1% formic acid (Supplementary Materials and Methods). The purified peptides were quantified using a QStar Elite mass spectrometer (AB SCIEX, USA) and duplicate LC-MS/MS runs were conducted for each iTRAQ™ experiment (Supplementary Materials and Methods). The combined data were processed using the Paragon™ algorithm in ProteinPilot™ 3.0 software (AB SCIEX, USA; Supplementary Materials and Methods) and each MS/MS spectrum was compared to the *N. italica* R11 genome. Protein quantification was determined using the ratios of the areas under the mass spectrometry peaks at 114, 115, 116, and 117 Da (corresponding to the iTRAQ™ 4-plex labels bound to the peptides from each of the four biological samples). The following criteria were used to identify differentially expressed proteins: an unused protein score >1.3 (corresponding to a confidence limit of 95%), at least two unique peptides detected, and *p* < 0.05 (Student's *t*-test, assuming equal variance). Proteins were considered differentially expressed when detected with significance in at least two of the total three biological replicates. The number of differentially expressed proteins was then compared to the predicted proteome of *N. italica* R11 using IMG/ ER (Taxon ID 647533206; Markowitz et al., 2012). Differentially expressed proteins were reported as the GenBank accession numbers from the *Ruegeria* sp. R11 genome entry (NZ_DS999055) and the COG annotations for these translated gene entries were reported as given in the NCBI database (Altschul et al., 1990).

## Results

### *N. italica* R11 **Δ***varR* shows reduced virulence against *D. pulchra* and is impaired in biofilm formation

**Figure 1:**
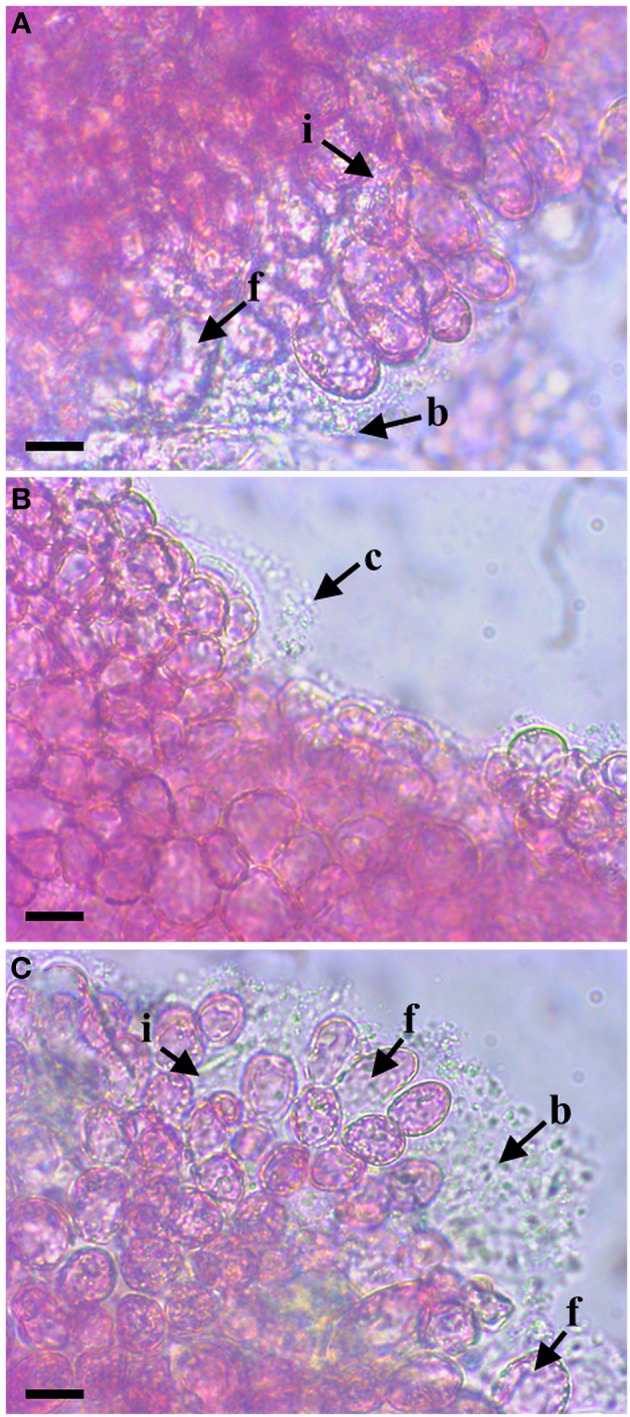
**Representative micrographs of ***D. pulchra*** sporelings inoculated with ***N. italica*** R11 WT (A), Δ***varR*** (B), and the Δ***varR*** strain complemented with WT ***varR*** (CΔ***varR***) (C)**. Sporelings inoculated with WT or CΔ*varR* bacteria show signs of infection and disease consistent with previous reports (Case et al., 2011). These include damage and bleaching/fading of algal cells (as denoted by f), the presence of a thick biofilm (as denoted by b), and the invasion of bacteria within algal tissue and/or algal cells (as denoted by i) as observed in a and c. The *varR* allelic exchange mutant **(B)** exhibited no capacity to cause bleaching or damage to the algal cells, nor was there evidence of bacteria invading the algal tissue or cells (across the triplicate experiments). Moreover sporelings inoculated with Δ*varR* **(B)** were relatively free from bacterial biofilms with only the occasional thin biofilm being observed **(C)**. Scale bar = 10 μm.

In order to investigate the role of *varR* in the virulence of the macroalgal pathogen *N. italica* R11, an allelic replacement mutant was generated and subsequently tested in an *in vivo* infection assay for *D. pulchra*. *N. italica* R11 WT formed a thick biofilm on chemically undefended *D. pulchra* thalli, invasion of algal tissue and/or cells and evidence of algal cell damage and bleaching (Figure 1A, Supplementary video 1), consistent with previous observations (Case et al., 2011). For *D. pulchra* sporelings inoculated with the *N. italica* R11 Δ*varR* strain, biofilm formation on the surface of the sporelings was reduced and no tissue invasion or bleaching was observed in any of the replicates (Figure 1B, Supplementary video 2). This outcome was not due to polar effects of the knock-out as the WT phenotypes of invasion, biofilm formation, and tissue damage were recovered in the trans-complemented strain CΔ*varR* carrying the plasmid pBBR1-varR_wt (Figure 1C, Supplementary video 3). These data suggest that VarR regulates the virulence of *N. italica* R11 in chemically undefended *D. pulchra*.

**Figure 2:**
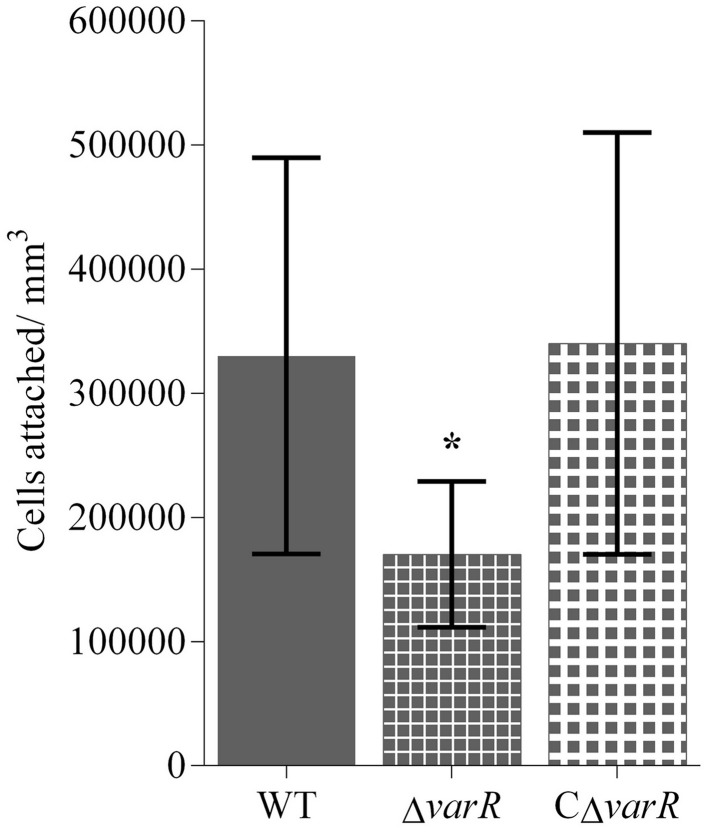
**Attachment to κ-carrageenan for ***N. italica*** R11 Δ***varR*** (small squares) compared to WT (solid fill) and CΔ***varR*** (large squares) after 6 h incubation**. The number of cells attached per mm^3^ was determined using direct counts of attached bacteria in a Helber bacterial counting chamber. Error bars represent standard deviation, *n* = 9. Significance was assessed using an ANOVA. Attachment of Δ*varR* was significantly reduced compared to both WT and CΔ*varR* (*p* < 0.05) as denoted by ^*^.

Previous reports have proposed that colonization is an important virulence factor for *N. italica* R11 (Case et al., 2011), and given the impaired pathogenicity exhibited by *N. italica* R11 Δ*varR*, we investigated surface attachment and biofilm formation in this strain. We first analyzed the attachment of *N. italica* R11 Δ*varR* to the sulfated polysaccharide κ-carrageenan, which is an abundant biological polymer in the cell wall matrix of red macroalgae (Fredericq et al., 1996). When compared to the WT strain, the *N. italica* R11 Δ*varR* mutant exhibited a significantly reduced capacity (*p* < 0.05) to adhere to κ-carrageenan coated surfaces after 6 h (Figure 2). Furthermore, the WT phenotype was restored when the Δ*varR* strain was complemented (i.e., strain CΔ*varR* vs. WT; *p* > 0.05; Figure 2). These results show that VarR contributes to the adhesion of *N. italica* R11 to biological polymers likely encountered during the colonization of *D. pulchra*.

**Figure 3:**
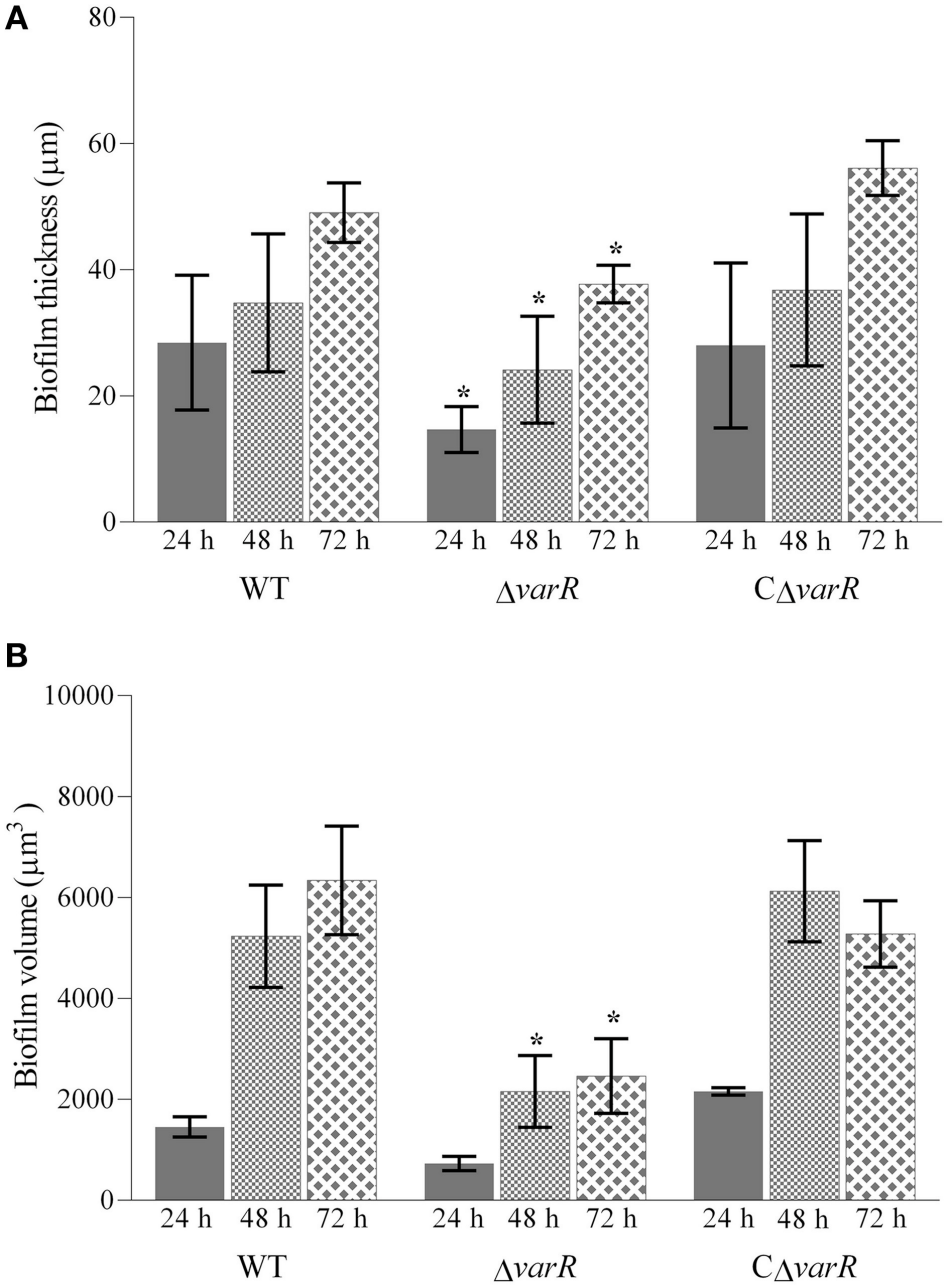
**Characterization of biofilm thickness (A) and volume (B) for ***N. italica*** R11 WT, Δ***varR***, and CΔ***varR*** at 24 h (solid fill), 48 h (small diamonds), and 72 h (large diamonds) post inoculation on a glass surface**. Quantification of biofilm characteristics was performed for Z-stack images using IMARIS software and the data analyzed using a PERMANOVA. Error bars represent standard deviation, *n* = 45; ^*^ denotes a significant difference at the corresponding time point, *p* < 0.05.

Given the reduced attachment phenotype displayed by *N. italica* R11 Δ*varR*, we further investigated biofilm progression in this strain. Biofilms were established on glass slides within a continuous flow-through cell and visualized using Confocal Laser Scanning Microscopy every 24 h for 3 days. The biofilm characteristics of *N. italica* R11 Δ*varR* were different to that of the WT strain (Figure S1) with a significant reduction in biofilm thickness at each time point analyzed (Figure 3A, *p* < 0.05) and a significantly lower average biofilm volume after 48 h of growth (Figure 3B, *p* < 0.05). In contrast, the biofilm characteristics of *N. italica* R11 CΔ*varR* were indistinguishable from the WT (thickness in Figure 3A: *p* > 0.05 and volume in Figure 3B, *p* > 0.05) at any of the time points observed. Overall, the development of biofilms by *N. italica* R11 Δ*varR* was delayed compared to the WT, and VarR appears to be involved in mediating the formation of cellular aggregates during biofilm maturation in *N. italica* R11.

### The biofilm proteome for *N. italica* R11 WT

To obtain further insight into the molecular mechanisms that are important for colonization and virulence of the algal pathogen, we performed global expression analysis (proteomics) on surface associated *N. italica* R11. Mass spectrometry analysis identified 3038 unique proteins, corresponding to 83% of the predicted proteome of *N. italica* R11. As the gene *varR* likely encodes for a transcriptional regulator and the Δ*varR* strain displays reduced capacity to infect *D. pulchra* and form biofilms *in vitro*, we first determined the proteins that are generally important during surface-attached biofilm growth using iTRAQ™ labeling, and then investigated the proteins that are under the control of VarR.

**Figure 4:**
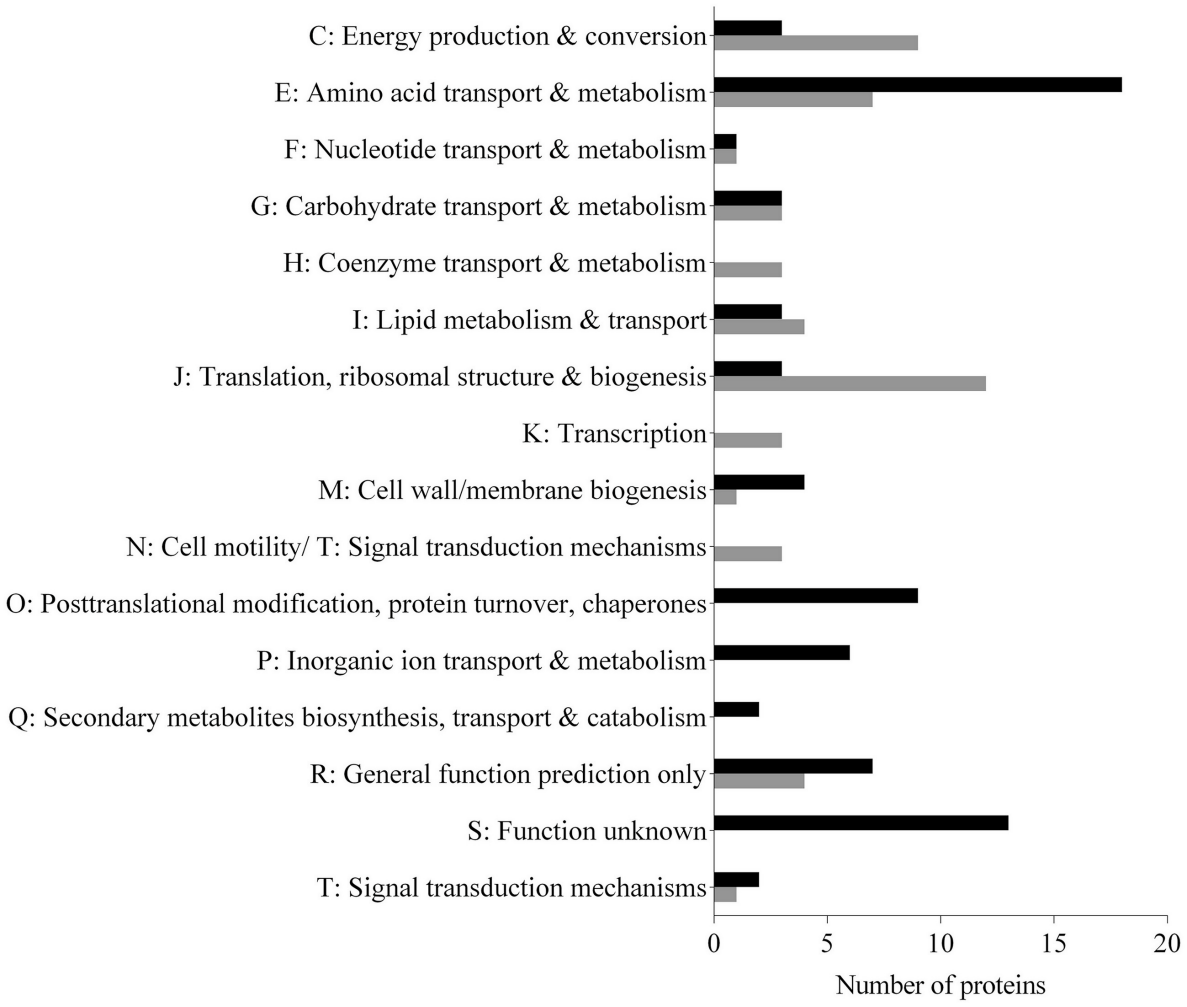
**Functional annotation of iTRAQ™-labeled proteins found to be up-regulated (black bars) or down-regulated (gray bars) in WT biofilm cells (WTB) compared to the planktonically grown cells (WTP) (Student's ***t***-test, ***p*** < 0.05)**. The GenBank accession number and annotation for each of the 125 proteins are given in Table S3. The COG categories were assigned to proteins based on annotation in the NCBI database.

The protein expression profile for *N. italica* R11 WT cells grown under biofilm conditions (WTB) was markedly different to that of the planktonically grown cells (WTP). A total of 125 proteins were differentially expressed in WTB compared to WTP (*p* < 0.05, Figure 4, Table S3). The differentially expressed proteins were assigned to a range of functions (Figure 4), with the majority of proteins up-regulated in biofilm assigned to Clusters of Orthologous Groups (COG) categories associated with nutrient uptake (COG E, I, P, and Q; e.g., EEB69622 and EEB69952), stress adaptation (COG O and P; e.g., EEB70501 and EEB7283) and protein turnover (COG O; e.g., EEB72870 and EEB71705; Table S3). In contrast, proteins involved in protein translation (COG J; e.g., EEB70057), cell motility (COG N; EEB71824), and signal transduction mechanisms (COG T; EEB70997) were generally down-regulated in biofilm cells, suggesting these functions are less important for biofilm cells (Table S3).

Eighteen percent of the differentially expressed proteins are categorized as either having a predicted function only (COG R, Figure 4) or exhibit no homology to characterized proteins (COG S, Figure 4; Table S3). All of the proteins assigned to COG S (uncharacterized) were up-regulated in wild type biofilm cells (WTB), suggesting that they may have a yet-unrecognized function in biofilm cells. A general trend was the abundance of ABC-type transporter proteins in biofilm relative to planktonic cells. In particular transporters for amino acids (e.g., EEB70113 and EEB72013), ions (EEB69252), secondary metabolites (EEB69518), and uncharacterized solutes (e.g., EEB72081 and EEB70354) were over represented in biofilm cells (Table S3).

The most differentially expressed protein identified in this study, EEB69472 (up-regulated 25-fold biofilm cells), has similarity (41%; with 20% protein identity) to the polyhydroxyalkanoate (PHA) associated (phasin) protein of *Cupriavidus necator* (formally *Ralstonia necator*) (AF314206). Other proteins with homology to factors involved in PHA metabolism were also up-regulated in WTB, including an acetyl-CoA acetyltransferase (EEB71680) and a PHA depolymerase (EEB71107), with 76.8 and 60.7% protein similarity to their respective *C. necator* proteins (AEI76812 and AEI79943).

### The *N. italica* R11 **Δ***varR* mutant exhibits differential protein expression relative to the WT

Having defined the “biofilm-associated” proteome for *N. italica* we next analyzed the proteome of the Δ*varR* strain grown under both planktonic and biofilm conditions relative to *N. italica* WT. Eighteen proteins assigned to COG categories associated with cellular biogenesis and metabolism were differentially expressed in planktonically grown Δ*varR* cells (ΔVP) compared to wild type planktonic (WTP) cells (Table S4). The Δ*varR* biofilm cells (ΔVB) showed 24 proteins differentially expressed relative to the WT biofilm counterparts (WTB) (Table S5). Five proteins were differentially expressed in Δ*varR* relative to the WT under both biofilm and planktonic growth conditions (Tables S4, S5 underlined accession numbers); including, for example the acetyl-CoA acetyltransferase (EEB71680) described above, which was down-regulated more than six-fold in Δ*varR* cells under both growth conditions relative to the WT.

The proteins with the highest fold increase in ΔVB relative to WTB were assigned to COG categories associated with protein translation (COG J), membrane integrity and transport (COG Q, M), and cell motility (COG N, T; Table S5). In contrast, the majority of proteins significantly down-regulation in ΔVB (and thus positively regulated by VarR) were annotated as factors involved in carbon, amino acid and lipid metabolism and ABC-type transport systems (COG C, E, I; Table S4). Moreover, several proteins down-regulated in ΔVB have no known homology or homology to uncharacterized proteins only (COG R, S; Table S5).

**Figure 5:**
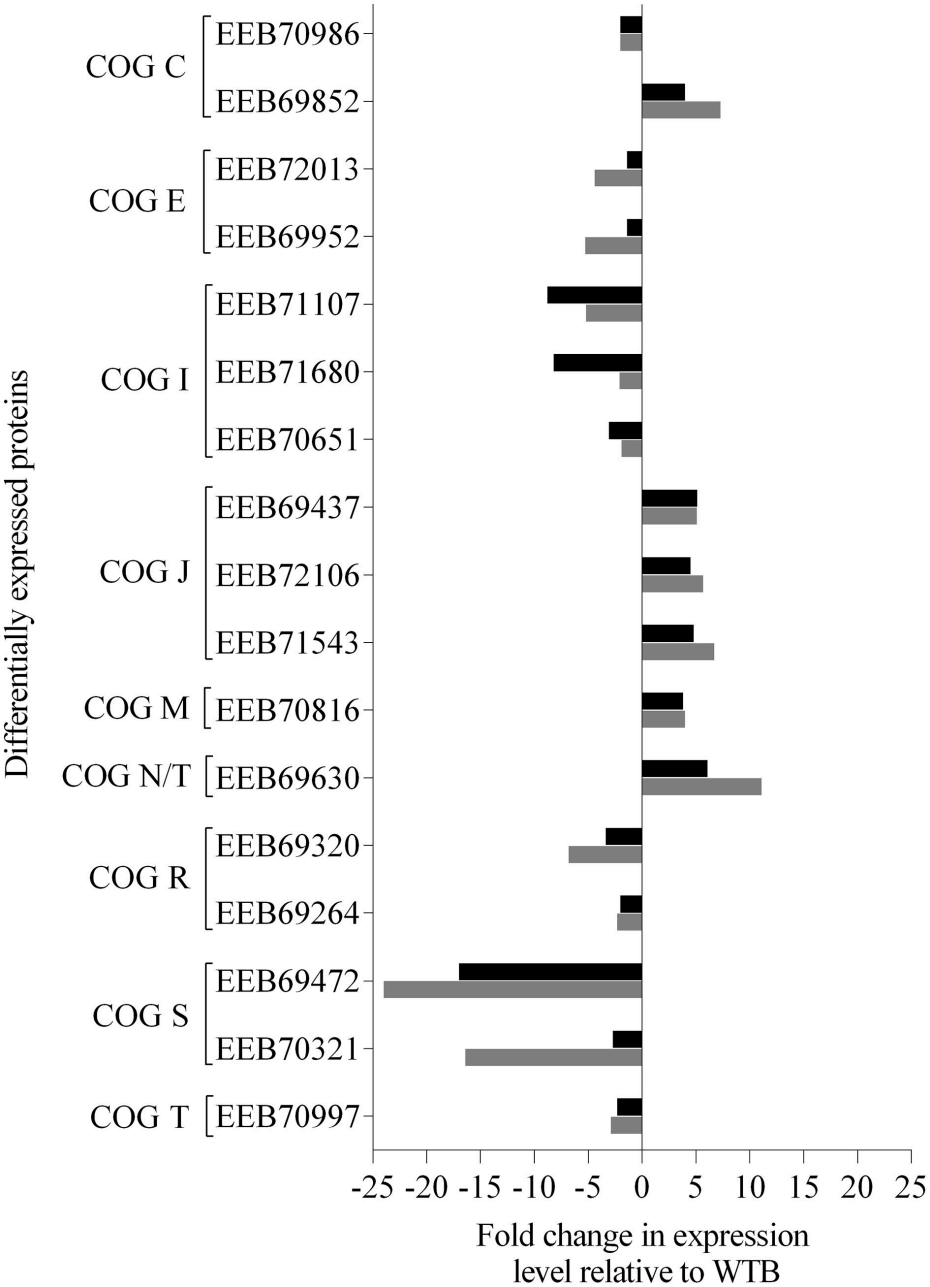
**Proteins both differentially regulated in the ***N. italica*** R11 biofilm proteome (Table S3) and also predicted to be regulated by VarR**. Seventeen of the proteins that were differentially expressed in Δ***varR*** biofilm cells (ΔVB) relative to the wild type (WTB) (Table S5: 11, up; 6, down) were also differentially expressed in WTB relative to WT planktonic cells (WTP) cells. The bars on the graph indicate the average fold change in either ΔVB relative to WTB (black bars) or WTP relative to WTB (gray bars) where the protein was detected in more than two biological replicates (Student's ***t***-test, ***p*** < 0.05). The respective protein expression value in WTB cells was employed as the baseline level to highlight a similar trend in expression in ΔVB and WTP. The GenBank accession number and Clusters of Orthologous Groups (COG) category are given for each protein. The COG categories are identified by letters as follows: C, energy production and conversion; E, amino acid transport and metabolism; I, lipid transport and metabolism; J, translation; M, cell wall/membrane/envelope biogenesis; N, cell motility; R, general function prediction only; S, function unknown; T, signal transduction mechanisms.

Analysis of the proteomic data for both Δ*varR* and WT revealed that 70% of the proteins differentially expressed in ΔVB compared to WTB (Table S5, denoted by ^∧^) were also detected in the WT biofilm proteome data (Figure 4, Table S3). Interestingly, these overlapping proteins showed the same relative trend in expression (up or down) in ΔVB and WTP when both were compared to WTB, suggesting they are controled by VarR under biofilm conditions (Figure 5). For example, 11 of the proteins that were down-regulated in ΔVB relative to WTB (Table S5) were also up-regulated in the WT under biofilm growth (Table S3) (Figure 5; down-regulated in WTP accordingly). These VarR-regulated proteins, include the PHA-associated proteins outlined above (EEB70651, EEB71680, EEB71107, and EEB69472), a putative periplasmic lipoprotein (EEB70321), proteins involved in amino acid transport and metabolism (EEB69952 and EEB72013), and a PrkA serine protein kinase (EEB70997; Table S5).

## Discussion

### The LuxR-type protein varR contributes to the colonization and virulence of *N. italica* R11

Here we show that the mutation of a *luxR*-type gene in *N. italica* R11, termed *varR*, reduces the capacity of this bacterium to infect *D. pulchra in vivo* (Figure 1), demonstrating a role for VarR as a virulence factor. Global regulatory mechanisms, such as QS, play a key role in the coordinated expression of virulence genes in mammalian and plant systems, where they control features such as toxin and exoenzyme production, motility, colonization, and biofilm development (de Kievit and Iglewski, 2000; Antunes and Ferreira, 2009; Ham, 2013). Here we add to this knowledge by demonstrating that, similar to QS regulation of virulence in animal- or phyto-pathogenic bacteria (von Bodman et al., 2003; Lang and Faure, 2014), a *luxR*-type protein contributes to the pathogenesis of a seaweed (macroalgal) pathogen. It should be noted however that whilst VarR does contain an AHL-binding domain (Fernandes et al., 2011), solo LuxR proteins from other bacteria have been shown to respond to host metabolites (González et al., 2013; Patel et al., 2013). Thus, future studies should also consider the influence of *D. pulchra* metabolites in mediating virulence of *N. italica* R11.

Our results show that loss of VarR function in *N. italica* R11 reduces the pathogens ability to attach to κ-carrageenan, a typical surface polymer of red macroalgae (Fredericq et al., 1996), and to form biofilms under laboratory flow cell conditions. Reduced pathogenicity of *N. italica* R11 Δ*varR* may thus be the result of a decreased capacity of this strain to successfully colonize algal surfaces due either to an impaired interaction with surface biopolymers and/or the transition to a biofilm life-style. These findings are in line with previous observations that colonization by *N. italica* R11 is a key stage in pathogenesis of *D. pulchra* (Campbell et al., 2011; Case et al., 2011). In other bacterial pathogens, LuxR-type regulators also function as virulence determinants by facilitating the transition from a planktonic to a sessile (biofilm) lifestyle in the presence of a susceptible host (de Kievit and Iglewski, 2000; Joo and Otto, 2012; Ham, 2013). These regulatory proteins specifically facilitate the coordinated expression of proteins that mediate the discrete stages of colonization, including attachment and biofilm maturation (Parsek and Singh, 2003; Koutsoudis et al., 2006; Dickschat, 2010), all of which are likely important factors for a pathogenic interaction between *N. italica* R11 and *D. pulchra*.

### *N. italica* R11 exhibits differential protein regulation under biofilm growth

Comparative and quantitative proteomics was used to provide insight into the molecular factors that facilitate biofilm formation in *N. italica* R11 and identified a subset of biofilm-associated proteins that are (directly or indirectly) regulated by VarR. The proteomic data demonstrated that 3.4% of the predicted proteome of *N. italica* R11 is differentially expressed between planktonic and biofilm conditions. This is consistent with other studies where between 1 and 9% of the predicted proteome is differentially expressed under biofilm conditions relative to planktonic growth (Whiteley et al., 2001; Schembri et al., 2003; de Souza et al., 2004; Mukherjee et al., 2011; Silva et al., 2011).

A general trend observed in the proteomic data was the up-regulation of proteins associated with substrate catabolism and nutrient acquisition, and the simultaneous down-regulation of factors responsible for protein translation and biogenesis, in WT biofilm cells (WTB) compared to cells grown planktonically (WTP). These data suggest that *N. italica* R11 biofilm cells have an altered biosynthetic and metabolic activity compared to planktonic cells, a finding that is consistent with other “omics” studies on bacterial biofilms (Waite et al., 2006; Shemesh et al., 2007; Lo et al., 2009; Clark et al., 2012).

The up-regulation of cellular transport factors, including numerous ABC-type transport proteins that facilitate the uptake of both organic (e.g., EEB72013, COG E), and inorganic (e.g., EEB70756, COG P) substrates, suggests that *N. italica* R11 cells are proficient at nutrient scavenging during biofilm growth (Figure 4). A predisposition for nutrient scavenging has been frequently reported for Roseobacter members (Moran et al., 2007; Christie-Oleza et al., 2012; Thole et al., 2012), and is interesting given that uptake and utilization of algal host metabolites was previously highlighted as a potential virulence factor in *N. italica* R11 based on genomic data (Fernandes et al., 2011). Moreover, the 10-fold overrepresentation of an ABC-type zinc/manganese/iron substrate binding protein (e.g., EEB70756 among other uptake factors; Table S3) suggests that *N. italica* R11 increases the expression of these transporters to cope with a potential metal ion limitation in the biofilm. A similar response has been reported for other bacterial pathogens, such as *Mycobacterium smegmatis* (Ojha and Hatfull, 2007; Monds and O'Toole, 2009).

Stress resistance has an important role in the persistence of biofilm cells in many bacterial species (Branda et al., 2005; Seneviratne et al., 2012), and up-regulation of proteins involved in oxidative stress resistance are frequently observed for biofilm cells (Tremoulet et al., 2002; Beloin et al., 2004; Shemesh et al., 2007; Pham et al., 2010; van Alen et al., 2010; Giaouris et al., 2013). Similarly, cellular detoxification and stress resistance enzymes, such as superoxide dismutase (SOX) (EEB72831), DsbA oxidoreductase (EEB70350), and a peroxiredoxin (EEB70501), were over-represented in the *N. italica* R11 biofilm proteome (Table S3). Antioxidant proteins facilitate the detoxification of reactive oxygen species (ROS), and the 15-fold increase in SOX observed in wild type biofilm (WTB) cells suggests an increase in ROS within biofilms that is likely due to limited gas diffusion (Costerton et al., 1995; Stewart and Franklin, 2008; Seneviratne et al., 2012). Recent work has further demonstrated a role for an antioxidant enzyme, glutathione peroxidase (GpoA) in the stress resistance and virulence of *N. italica* R11 (Gardiner et al., 2015), however this protein was not observed here to be differentially regulated in biofilm cells. It is possible that GpoA is either constitutively expressed or that this enzyme is only differentially expressed in response to specific growth conditions not tested here (i.e., host-associated conditions).

### varR controls the expression of a subset of biofilm-associated proteins in *N. italica* R11

LuxR-type proteins regulate the expression of traits involved in biofilm maturation and growth in a range of bacteria (Huber et al., 2001; Croxatto et al., 2002; Koutsoudis et al., 2006; Dickschat, 2010); and the data presented here suggests a similar role for VarR in *N. italica* R11. Quantitative proteomics demonstrated that the majority of proteins that are indirectly or directly under the control of VarR are also differentially expressed under biofilm conditions in *N. italica* R11 (Figure 5).

Chemosensory proteins, such as MCP's (methyl-accepting chemotaxis protein), facilitate chemotaxis, and the movement of motile cells toward external stimuli including surfaces (Wadhams and Armitage, 2004). A putative MCP (EEB69630) was negatively regulated by VarR, exhibiting the same relative trend in expression in the *varR* mutant biofilm cells (ΔVB) as in wild type planktonic (WTP) cells (Figure 5, Table S5), indicating that VarR suppresses the expression of this MCP during biofilm growth. In addition, the proteomics data (Figure 5, Tables S3–S5) show that VarR positively affects the expression of a putative serine protein kinase (PrkA-like protein) (EEB70997) that was also overrepresented in the biofilm proteome (Table S3). Serine protein kinase enzymes are important for bacterial surface colonization, particularly during biofilm maturation (Liu et al., 2011; Mikkelsen et al., 2011), and have a key role in the function of generalized two-component signal transduction (TCST) systems (Krell et al., 2010; Hunke et al., 2012). The observation that VarR regulates the expression of putative signal transduction proteins is in line with observations for other bacterial species such as *Pseudomonas aeruginosa*, where LuxR-type QS systems are linked to multiple regulatory mechanisms, including TCST (Ferrières and Clarke, 2003; Damron et al., 2012). Taken together, the available data show that VarR regulates that expression of traits that facilitate biofilm maturation as well as proteins with the potential to influence signal transduction and downstream gene expression.

This study identified that the majority of proteins, likely to be important for biofilm-associated growth in *N. italica* R11 are in fact (directly or indirectly) regulated by VarR (Figure 5). For example, the expression profile of numerous PHA-associated proteins in *varR* mutant biofilm cells (ΔVB) reflected the expression levels observed in planktonically grown WT cells (EEB69472, EEB71107, EEB70651, and EEB71680; Figure 5). Two of these proteins, a phasin family protein (EEB69472) and an acetyl-CoA acetyltransferase (EEB71680) appear to be positively regulated by VarR in *N. italica* R11 irrespective of growth conditions (Tables S4, S5). PHAs are bio-polyesters that accumulate in the cytoplasm of bacterial cells and provide protection against nutrient stress during biofilm growth by serving as readily accessible carbon and energy reserves (Pham et al., 2004; Campisano et al., 2008; Tribelli and Lopez, 2011; Berlanga et al., 2012; Escapa et al., 2012). LuxR-type regulators have been shown to regulate the expression of PHA-associated enzymes (Miyamoto et al., 1998) and proteins (Chambers et al., 2006), and have been found to be differentially expressed during the different stages of biofilm growth in *P. aeruginosa* (Campisano et al., 2008). Based on the available data, we propose that VarR regulates the accumulation and/or metabolism of PHA's in *N. italica* R11, and that the use of these bio-polyesters as energy reserves may be important for the survival and persistence of this pathogenic bacterium under the dynamic conditions likely encountered on its algal host surface.

In summary, the data presented here demonstrates a role for the *N. italica* R11 LuxR-type protein VarR in attachment, biofilm maturation, and infection of the red macroalga *D. pulchra*. Proteomics demonstrated that VarR controls the expression of a subset of biofilm-associated proteins involved in cellular functions, including nutrient scavenging, and could provide *N. italica* R11 with an advantage during growth and colonization of *D. pulchra*. Moreover, four of the proteins that are directly or indirectly regulated by VarR have no significant homology to previously uncharacterized proteins (Figure 5, Table S5: EEB69264, EEB69320, EEB70321, EEB69472), and these proteins constitute candidate novel virulence factors that warrant further investigation (e.g., with immunohistology or Western blot to define localization).

Despite decades of research of model human and plant pathogens, there is still little understanding of the factors that make a bacterium pathogenic (Brown et al., 2012; Casadevall and Pirofski, 2014; de Lorenzo, 2015). Here we show that the LuxR-type protein, VarR, that is unique to the Roseobacter strains capable of causing algal disease (Fernandes et al., 2011) acts to control important aspects of host colonization and virulence in *N. italica* R11. Intriguingly neither previous genomic (Fernandes et al., 2011) nor the current proteomic analysis have shown *N. italica* R11 to possess a unique set of bacterial virulence factors, which would be expected in the classical view of bacterial pathogenesis (Gal-Mor and Finlay, 2006; Jones and Oliver, 2009; Vrancken et al., 2013). Rather it would appear that the capacity for *N. italica* R11 to cause harm to its algal host is mediated at the level of gene regulation of several functions, likely in response to environmental or host conditions. This perspective is consistent with a contemporary view of pathogenesis that identifies virulence to be more complex than the presence of specific bacterial determinants (Casadevall and Pirofski, 2001, 2002), and as exemplified in a recent study of the pathogen *P. aeruginosa* where neither the origin of the strain (i.e., environmental or medical) nor the predominance of known virulence traits in the genome were found to be predictors of the ability of a particular strain to cause disease (Hilker et al., 2015).

To conclude, the pathogen–host interaction between *N. italica* R11 and *D. pulchra* provides a model framework of virulence in bacterial pathogens that is influenced by the environmental context (i.e., host defense capacity) and ability of the pathogen co-ordinate gene expression (i.e., via VarR). This model not only provides insight into the factors that may be contributing to the decline of macroalgal species in the context of a changing environment, but also enhances understanding of the global factors that mitigate microbial disease in marine ecosystems.

### Conflict of interest statement

The authors declare that the research was conducted in the absence of any commercial or financial relationships that could be construed as a potential conflict of interest.
