# Supplementary material for: VarR controls colonization and virulence in the marine macroalgal pathogen Nautella italica R11
Source: Front Microbiol. 2015 Oct 13;6:1130. doi: 10.3389/fmicb.2015.01130 (PMC4602140; doi:10.3389/fmicb.2015.01130)
Supplement: Supplementary file 4 [file DataSheet1.PDF]

**Gardiner *et al.* Supporting Material**

## Supplementary Materials and Methods

Mutagenesis of *varR* in *N. italica* R11

A *N. italica* R11 *varR* allelic replacement mutant strain, Δ*varR* was constructed using the Splicing by Overlap Extension PCR (SOE-PCR) strategy (Horton, 1995), coupled with bi-parental conjugation (Thoma, 2009) and homologous recombination of the SOE-PCR fragment with the genome of the recipient bacterium. The SOE-PCR strategy involved the amplification of two 1000 bp PCR products that targeted the *varR* gene; the varUpF/R primers (Table S2) targeted the first half of the *varR* gene ('1'), and the remaining *varR* gene sequence ('2') was amplified using the varDnF/R primers, with the deliberate exclusion of a 100 bp region from within the gene. A chloramphenicol resistance marker was separately amplified from the plasmid vector pBBR1 MCS (Kovach et al., 1995) using primers targeting the chloramphenicol acetyltransferase (*cat*) gene (Table S2). The varUpR and varRDnF primers include 5' overhang sequences that corresponds to the complement sequences of the primers used to amplify the *cat* gene (Table S2). A recombinant *varR* knockout fragment was constructed by 'PCR splicing' (Horton, 1995) the 5'overhangs on the '1' and '2' PCR products onto the 5` and 3` ends of the *cat* PCR product. Specifically, the PCR products termed '1', '2' and 'cat' were pooled in a SOE-PCR reaction that involved 5 PCR cycles without primers to align the fragments followed by addition of the primers varUpF and 'varDnR for 25 cycles to amplify the entire *varR* knockout fragment. Following PCR product purification (Zymo Research Corporation, USA), the knockout fragment was phosphorylated using T4 Polynucleotide Kinase (New England Biolabs Inc) and ligated into a broad host range suicide vector pKmobG11 (Katzen et al., 1999) that had been treated with BamH1, Large (Klenow) Fragment DNA Polymerase I and Antarctic Phosphatase (New England Biolabs Inc). The suicide vector containing the *varR* knockout cassette was then delivered to strain R11 via an *E. coli* ST18 donor strain using bi-parental conjugation (Thoma, 2009). Putative strain R11 Δ*varR* exconjugants were selected for on half-strength marine agar plates supplemented with chloramphenicol and 50 μg ml-1 of 5-bromo-4-chloro-3-indoxyl-b-D-glucuronide (x-gluc). Putative conjugants were confirmed by colony PCR using primers varUpF and FlankR (Table S2), which amplified the respective gene knockout construct and a region on the chromosome of strain R11 adjacent to the knockout construct.

Complementation of the *varR* gene replacement knockout mutant

The WT *varR* gene was amplified by PCR using primers varUpF and varDnR, which target regions 1000 bp upstream and 1000 bp downstream of the gene, respectively, and therefore include potential upstream promoter regions or downstream terminator regions. The purified PCR product was cloned into the SmaI restriction site of the broad-host range plasmid vector pBBR1 MCS-5 (Kovach et al., 1995) using standard procedures (Ausubel et al., 1999), and transferred to Δ*varR* via bi-parental conjugation with the donor strain *E. coli* ST18 (Thoma, 2009). Putative conjugants were selected for on half-strength marine agar plates containing gentamicin and chloramphenicol and verified by PCR using 'UpF' and a reverse primer that targeted the gentamycin resistance cassette on the plasmid vector pBBR1 MCS-5 (Kovach et al., 1995) (Table S2).

Quantitative mass spectrometry (LC MS/MS) with Isobaric Tags for Relative & Absolute Quantitation (iTRAQ™) labelling

Protein concentrations for the crude protein extracts obtained from the biofilm and planktonic cells were determined using the 2D Quant assay (GE Healthcare, UK) according to the manufacturer's protocol. To provide a qualitative assessment of the sample quality, the proteins were separated using SDS-PAGE on a Mini-PROTEAN® TGX™ Precast Gel (Bio-Rad, USA) and stained with SimplyBlue™ SafeStain (Invitrogen, USA). One hundred micrograms of each of the crude protein extracts were reduced and the cysteines blocked before trypsinization with 5 µg of sequencing grade porcine modified trypsin (Promega, Madison, USA). LC MS/MS analysis was conducted to test the efficiency of digestion before labelling.

The peptides were labelled with the iTRAQ™ reagents according to manufacturer's instructions (AB SCIEX, Foster City, USA). Briefly, the iTRAQ™ Reagent vials were dissolved in absolute ethanol and spun down, before addition of the four reagents to the respective samples. Following 2 h incubation at room temperature, the labelled peptide samples were combined as one set and unbound iTRAQ™ reagents, trypsin and SDS were removed by strong cation exchange (SCX) chromatography using an ICAT® Cation Exchange Cartridge using an Opti-Lynx cartridge holder (AB SCIEX, USA) and a syringe pump (KD Scientific, USA) at a flow rate of 10 ml $h^{-1}$ according to the manufacturer's instructions. Desalting was performed with an Oasis HLB Plus Light Cartridge (Waters Corporation, USA) at an injection rate of 10 ml $h^{-1}$ as per the manufacturer's directions. The

eluent was vacuum-dried and the peptide pellets dissolved in 0.05% HFBA (heptafluorobutyric acid)/1% formic acid.

The solubilized peptides were chromatographed twice to provide two technical replicates by online strong cation exchange (SCX) and nano-C18 LC using an Ultimate HPLC with a Switchos and Famos autosampler system (LC-Packings, Netherlands). Peptide samples were loaded onto a small SCX micro trap (~500 micron × 12 mm, Poros S10, AB SCIEX, USA) and were eluted sequentially with 5, 10, 15, 20, 25, 30, 40, 50, 100, 250, 500, and 1,000 mM ammonium acetate (20 µl). The initial unbound fraction and each salt step fraction were concentrated and desalted onto a micro-C18 pre-column (500 µm × 2 mm; Michrom Bioresources, USA) at 15 µl $min^{-1}$. After a 10 min wash the pre-column was switched (Valco 10 port valve, Dionex) in line with a fritless nano-analytical column (75 µm × ~10 cm) containing C18 reverse phase media (5 µm, 200 Å, Magic, Michrom Bioresources, USA) eluted with a linear gradient of ACN (acetonitrile) in 0.1% (v/v) formic acid from 2% to 36% ACN for 74 min, followed by 1 min at 90% ACN at ~300 nl/min. The column was connected via a fused silica capillary to a low-volume tee (Upchurch Scientific, USA) where high voltage (2,300 V) was applied and the column tip was positioned ~1 cm from the orifice of a QStar Elite mass spectrometer (AB SCIEX, USA). The QStar was operated in information-dependent acquisition mode. A time-of-flight (TOF) MS survey scan was acquired (m/z 350-1750). The three most abundant multiply charged ions (counts > 25) were sequentially selected by the quadrupole for TOF MS/MS analysis. Tandem mass spectra were accumulated for up to 2.5 s (m/z 65-2,000) using the Advanced IDA parameters automatic MS/MS accumulation (set to 20) and automatic collision energy.

The search parameters in the ProteinPilot™ 3.0 software (AB SCIEX, USA) were set to: sample type = iTRAQ™ 4-plex (peptide labelled); cysteine alkylation = iodoacetamide; digestion = trypsin; identification focus = biological modifications; search effort = thorough ID. To correct for small differences in protein loading, the generated ratios of all detected proteins were normalized by bias correction with the ProteinPilot™ software.

Table S1: Bacterial strains and plasmids used in this study

| **Strain or plasmid** | **Relevant genotype** | **Reference** |
|---|---|---|
| ***N. italica* R11** | | |
| WT | Wild type strain | Case et al. (2011) |
| Δ*varR* | *varR* mutant: Δ*varR*::*Cat Kan* | This study |
| CΔ*varR* | Complemented *varR* mutant; Δ*varR*::*Cat Kan*; pBBR1-varR_wt, *Gen* | This study |
| ***E. coli*** | | |
| *E. coli DH5-α* | supE44 *hsdR17 recA1 endA1 gyrA96* thi-1 | |
| ST18 | Δ*hemA* TpR *recA*, thi, pro, *hsdR*-M+RP4: 2-Tc:Mu: *Kan* Tn7 *λpir* | Thoma (2009) |
| **Plasmids** | | |
| pKmobGII | 5.9 kb broad host range mobilizable suicide vector, R6K *ori mob gusA Kan* | Katzen et al. (1999) |
| pKmobGII varR | Knock-out construct of *varR Kan Cat* | This study |
| pBBR1 MCS5 | 4.72 kb broad host range mobilizable vector, *rep mob Gen* | Kovach et al. (1995) |
| pBBR1-varR_wt | Complementation vector containing intact *varR, Gen* | This study |

#*Cat*, chloramphenicol resistance; *Kan*, kanamycin resistance; *Gen*, gentamycin resistance; *λ pir* (a lambda prophage *pir* gene), π protein for plasmid replication of the R6K *ori*; *gusA*, beta-glucuronidase

Table S2: Primers used in this study to generate and confirm the *N. italica* R11 strains, Δ*varR* and C Δ*varR*. The 5' overhang sequences on the 'UpR' and 'DnF' primers that align with the CmF and CmR primers are given in italics.

| **PCR product** | **Primer pair** | **Sequence 5'- 3'** | **Amplicon size (bp)** |
|---|---|---|---|
| *cat* | CmF/CmR | GCTGCATTAATGAATCGGCCA/ GAATAAATACCTGTGACGGAAGATCACTTC | 903 |
| '1' | varUpF/ varUpR | ATCGCCTTTCAAACCAATCT/ *TGGCCGATTCATTAATGCAGC*CATCACCTCGAAGACCAACC | 1090 |
| '2' | varDnF/ varDnR | *GAAGTGATCTTCCGTCACAGGTAATTATTC*GCCTTTAGTCCGTGGTTCAG/ CAACCGCATTCCAAGTAACC | 923 |
| *varR* knockout fragment | varUpF/ varRDnR | ATCGCCTTTCAAACCAATCT/ CAACCGCATTCCAAGTAACC | 2916 |
| Δ*varR* confirmation | varUpF/ FlankR | ATCGCCTTTCAAACCAATCT/ CATCCAGTGTTTTGGGCTTT | 3025 |
| CΔ*varR* confirmation | GenF/ varDnF | GACGCACACCGTGGAAA/ GAAGTGATCTTCCGTCACAGGTAATTATTCGCCTTTAGTCCGTGGTTCAG | 2238 |

**Supplementary Results**

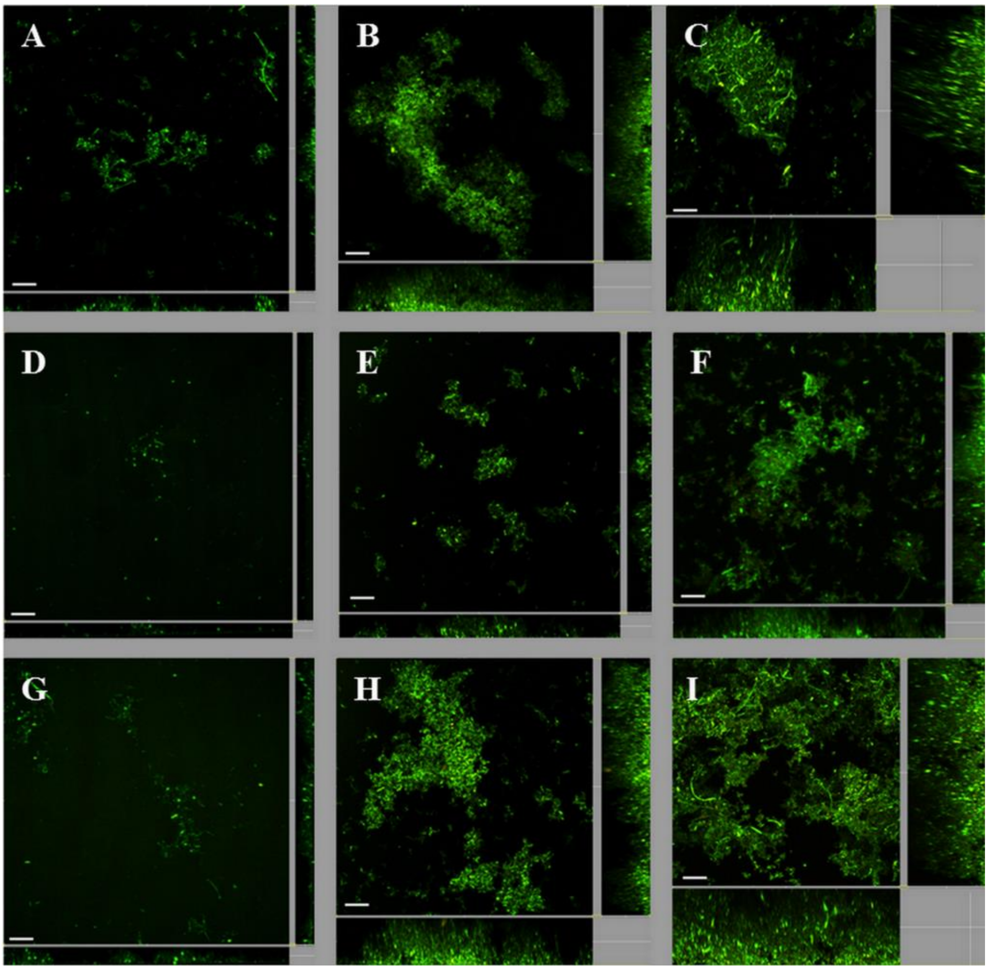


Figure S1: Confocal Microscopy images showing typical biofilm formation for *N. italica* R11 WT (A B C), Δ*varR* (D E F), and CΔ*varR* (G H I) after 24 h (A D G), 48 h (B E H), and 72 h (C F I) growth. Bacteria are stained with LIVE/DEAD® BacLight™ (Molecular Probes): live cells are fluorescing green; dead cells are red; cells that are in the process of dying are yellow. Z-stack images were acquired using an Olympus Fluoview FV1000 Confocal Laser Scanning Microscope and processed using IMARIS software. Scale bar = 20μm

Table S3: Proteins that were significantly differentially expressed in *N. italica* R11 WT cells under biofilm growth (WTB) compared to planktonic conditions (WTP). Proteins were detected with $p < 0.05$ across two biological replicates, except where * denotes the protein was identified in all three biological replicates. Shading denotes proteins that were down regulated in expression in WTB relative to WTP. The Genbank accession number for the proteins is given along with the protein description and COG category as determined in the NCBI database.

| Accession number | NCBI Protein Annotation | Fold change in expression |
|---|---|---|
| | *C: Energy production and conversion* | |
| EEB72409 | Malate synthase G | 6.9 |
| EEB71438 | Aconitrate hydratase 1 | 4.9 |
| EEB70986 | Pyruvate carboxylase | 2 |
| EEB72091 | 2-oxoglutarate dehydrogenase, E1 component | -2.3 |
| EEB71804 | Indolepyruvate ferredoxin oxidoreductase | -2.4 |
| EEB72637 | Flavodoxin oxidoreductase, gamma subunit protein | -2.6* |
| EEB72898 | Phenylacetic acid degradation protein, PaaN | -3.3 |
| EEB72553 | Proline dehydrogenase family protein | -5.4* |
| EEB72453 | NADH-quinone oxidoreductase, G subunit | -5.4 |
| EEB71249 | Aldehyde dehydrogenase family protein | -5.6* |
| EEB69852 | Aconitrate hydratase B | -5.7* |
| EEB70645 | Methylmalonic acid dehydrogenase | -7.3* |
| | *E: Amino acid transport and metabolism* | |
| EEB72454 | Peptidase family M3 protein | 7.3* |
| EEB70871 | Aminotransferase class IV | 7.1 |
| EEB70400 | Ornithine cyclodeaminase | 6.1* |
| EEB69952 | Arginase | 5.9* |
| EEB70113 | ABC-type glycine transporter, substrate-binding | 4.8* |
| EEB72013 | ABC-type peptide transporter, substrate-binding | 4.5* |
| EEB71507 | Gamma-glutamyltranspeptidase | 4.2* |
| EEB69826 | Peptidase family M20 protein | 3.7 |
| EEB70711 | L-threonine 3-dehydrogenase | 3.6* |
| EEB72079 | Leucyl aminopeptidase | 3.6* |
| EEB72156 | Oligoendopeptidase F | 3.5 |
| EEB71087 | Extracellular solute-binding proteins, family 5 | 3.5 |
| EEB71181 | Peptidase T | 3.4 |
| EEB69706 | Aspartate-semialdehyde dehydrogenase | 3 |
| EEB69622 | Glutamine synthetase, type I | 2.9* |
| EEB72235 | ABC-type oligopeptide transporter, periplasmic component | 2.8 |
| EEB71218 | Cystathionine gamma-synthase like enzyme | 2.3 |
| EEB71049 | UDP-N-acetylglucosamine 1-carboxyvinyltransferase | 2.2* |

| | | |
|---|---|---|
| EEB72864 | 2-isopropylmalate synthase | -2.1 |
| EEB71458 | ABC-type putrescine transporter, periplasmic component | -2.3 |
| EEB70780 | ABC-type amino acid transporter, periplasmic component | -2.6 |
| EEB72886 | 3-phosphoglycerate dehydrogenase | -2.9* |
| EEB72547 | Twin-arginine translocation pathway signal | -3.2* |
| EEB70354 | Extracellular solute-binding proteins, family 5 | -4.7 |
| EEB69263 | Extracellular ligand-binding receptor | -7.9 |
| | *F: Nucleotide transport and metabolism* | |
| EEB71027 | 5'-nucleotidase | 3.5* |
| EEB72523 | Phosphoribosyl transferase domain protein | -4.1 |
| | *G: Carbohydrate transport and metabolism* | |
| EEB72081 | Extracellular solute-binding protein, family 1 | 2.7* |
| EEB69962 | HAD-superfamily subfamily IIA hydrolase | 2.6 |
| EEB70292 | Triose-phosphate isomerase | 2.4 |
| EEB70779 | ABC-type sugar transporter, ATPase component | -2.4 |
| EEB69824 | Extracellular solute-binding protein, family 1 | -5.4* |
| EEB69330 | Extracellular solute-binding protein, family 7 | -6.7 |
| | *H: Coenzyme transport and metabolism* | |
| EEB72422 | S-adenosylmethionine synthetase | -2.7* |
| EEB72820 | Aminotransferase class III | -2.2 |
| EEB71985 | Aminotransferase class III | -8.6 |
| | *I: Lipid metabolism and transport* | |
| EEB71107 | Polyhydroxyalkanoate depolymerase, intracellular | 5.3 |
| EEB71688 | 3-hydroxyisobutyrate dehydrogenase | 3.1 |
| EEB71680 | Acetyl-CoA acetyltransferase | 2.1 |
| EEB70651 | Acetoacetyl-CoA reductase | 1.9 |
| EEB72443 | Methylmalonyl-CoA mutase | -2.7 |
| EEB72740 | Acetyl-CoA carboxylase, carboxyltransferase component | -3.2 |
| EEB72812 | Acyl-CoA dehydrogenase | -3.9 |
| EEB72289 | Acetyl/propionyl-CoA carboxylase, alpha subunit | -4.6 |
| | *J: Translation, ribosomal structure and biogenesis* | |
| EEB71968 | AsnC family transcriptional regulator | 6.5 |
| EEB72503 | Glutathione synthetase | 3.2 |
| EEB69693 | Alanyl-tRNA synthase | 1.9 |
| EEB71499 | Translation elongation factor Ts | -2.6 |
| EEB70221 | Ribosomal protein L7/L12 | -3.5 |
| EEB72731 | Ribosomal protein S10 | -3.8 |
| EEB72466 | Translation elongation factor G | -4.2* |
| EEB71716 | Ribosome-associated protein Y | -4.3 |
| EEB69437 | Ribosomal protein L3 | -4.7 |
| EEB72506 | Ribosomal protein S2 | -5.6 |
| EEB72106 | Ribosomal protein S1 | -5.7 |
| EEB71543 | Ribosomal protein L15 | -6.3* |

| | | |
|---|---|---|
| EEB72511 | Ribosomal protein L9 | -6.7 |
| EEB70057 | Ribosomal protein L1 | -7.3* |
| EEB72021 | Ribosomal protein L6 | -9 |
| | *K: Transcription* | |
| EEB72887 | Putative cold-shock DNA-binding domain protein | -3.5 |
| EEB69671 | MarR family transcriptional regulator | -11.4 |
| | *M: Cell wall/membrane biogenesis* | |
| EEB70840 | Outer Membrane Protein, OmpA | 14.3 |
| EEB70168 | ABC-type/ RND efflux pump, membrane efflux protein | 6.5* |
| EEB72598 | Glutamine-fructose-6-phosphate transaminase | 4.0* |
| EEB70281 | Lytic murein transglycosylase | 2.1 |
| EEB70816 | Outer membrane protein assembly complex, YaeT protein | -3.7 |
| | *N: Cell motility/ T: Signal transduction mechanisms* | |
| EEB71824 | Methyl-accepting chemotaxis sensory transducer | -6.9* |
| EEB69311 | Histidine kinase | -9.8* |
| EEB69630 | Methyl-accepting chemotaxis sensory transducer | -11.1* |
| | *O: Posttranslational modification, protein turnover, chaperones* | |
| EEB70350 | DsbA oxidoreductase | 9.8 |
| EEB71705 | Periplasmic serine protease, DO/DeqQ family protein | 9.6* |
| EEB70501 | Peroxiredoxin | 9.6 |
| EEB72280 | Thiol-disulfide interchange protein, DsbA family | 9.4* |
| EEB71775 | FeS assembly protein SufB | 5.2 |
| EEB72892 | ATP-dependent protease La | 3.9* |
| EEB70461 | ATP-dependent metallopeptidase, HflB subfamily | 2.8* |
| EEB69723 | ATP-dependent Clp protease, ATP-binding subunit | 2.5 |
| EEB72870 | Chaperonin GroEL | 1.6 |
| | *P: Inorganic ion transport and metabolism* | |
| EEB72831 | Superoxide dismutase | 15.2* |
| EEB70756 | ABC-type zinc/manganese/iron transporter, periplasmic | 10.8* |
| EEB69747 | Extracellular solute-binding protein, family 1 | 10.2 |
| EEB69398 | TonB-dependent receptor, plug | 4.5* |
| EEB71350 | Toxic anion resistance protein | 2.9 |
| EEB69252 | ABC- type transporter, periplasmic hemin-binding protein | 2.3 |
| | *Q: Secondary metabolites biosynthesis, transport and catabolism* | |
| EEB69518 | ABC-type organic solvent transporter, auxiliary component | 12.4* |
| EEB71612 | Extracellular solute-binding protein, family 7 | 3.1 |
| | *R: General function prediction only* | |
| EEB70957 | Imelysin-like peptidase | 7.6 |
| EEB69320 | Alpha/beta hydrolase family | 7.4 |
| EEB72784 | TRAP transporter solute receptor, TAXI family | 7.3 |

| | | |
|---|---|---|
| EEB71080 | D-beta-hydroxybutyrate dehydrogenase | 6.3 |
| EEB71038 | Cobaltochelatase, CobS subunit | 4.6* |
| EEB72449 | Amidohydrolase family protein | 3.7 |
| EEB70327 | Amidase | 2.4 |
| EEB69264 | Predicted epimerase, PhzC/PhzF homolog | 2.3 |
| EEB71376 | 3-hydroxyacyl-CoA dehydrogenase, type II | -2.3 |
| EEB72893 | Predicted nucleoside-diphosphate-sugar epimerase | -4.5 |
| EEB70264 | Uncharacterized periplasmic binding protein domain | -7.2 |
| | *S: Function unknown* | |
| EEB69472 | Phasin family protein | 25.6* |
| EEB71183 | Hypothetical | 21.7* |
| EEB70321 | Predicted periplasmic protein | 15.8* |
| EEB70310 | Hypothetical | 15.4 |
| EEB70606 | Predicted outer membrane protein | 15.1* |
| EEB70121 | Hypothetical | 9.9 |
| EEB70107 | Hypothetical | 7.9* |
| EEB69525 | Hypothetical | 7.6* |
| EEB70709 | Predicted periplasmic lipoprotein | 7.5 |
| EEB72259 | Universal stress protein family protein | 4.5 |
| EEB70593 | Hypothetical | 4 |
| EEB72283 | Hypothetical | 3.4 |
| | *T: Signal transduction mechanisms* | |
| EEB72578 | Response regulator containing CheY-like receiver, AAA-type ATPase, and DNA-binding domains | 4.2 |
| EEB70997 | Serine protein kinase, PrkA | 3.0* |
| EEB69307 | Anti-sigma-factor antagonist | 2.8 |
| EEB69994 | GTP-binding protein, TypA/BipA | -1.9 |

Table S4: Proteins differentially expressed in Δ*varR* planktonic cells (ΔVP) compared to WT planktonic cells (WTP). The average fold change in expression in ΔVP is shown, with grey shading indicating down-regulation. Proteins were detected with $p < 0.05$ across two biological replicates except where * denotes that were proteins identified in three biological replicates. Shading denotes proteins that were down regulated in expression in ΔVP relative to WTP. Underlined accession numbers indicate that the protein was also differentially expressed in Δ*varR* relative to WT under biofilm growth conditions (Table S5) Genbank accession numbers are given along with the COG category and protein description provided in the NCBI database.

| Accession number | NCBI Protein Annotation | Fold change in expression |
|---|---|---|
| | *C: Energy production and conversion* | |
| EEB69905 | ATP synthase F1, alpha subunit | 2.7 |
| | *E: Amino acid transport and metabolism* | |
| EEB70922 | Diaminopimelate/ornithine decarboxylase | 3.1 |
| EEB70354 | Bacterial extracellular solute-binding proteins, family 5 | -2.8* |
| EEB71221 | Bacterial extracellular solute-binding protein, family 8 | -2.3 |
| | *G: Carbohydrate transport and metabolism* | |
| EEB69628 | Oligo-1,6-glucosidase | 3* |
| EEB70046 | Alpha-glucosides-binding periplasmic protein | 2.2* |
| EEB72540 | Transketolase | 1.7 |
| | *I: Lipid transport and metabolism* | |
| EEB71680 | Acetyl-CoA acetyltransferase | -6.3* |
| | *J: Translation, ribosomal structure and biogenesis* | |
| EEB72430 | 3' exoribonuclease family protein | 3.6 |
| EEB72106 | Ribosomal protein S1 | 2.5 |
| EEB72021 | Ribosomal protein L6 | 2.1 |
| | *M: Cell wall/membrane/envelope biogenesis/ U: Intracellular trafficking, secretion, and vesicular transport* | |
| EEB72298 | Outer membrane efflux protein, TolC family | -2 |
| | *O: Posttranslational modification, protein turnover, chaperones* | |
| EEB72892 | ATP-dependent protease La | 3.1 |
| EEB70029 | Membrane protease subunits, stomatin/prohibitin homolog | -2.4 |
| | *P: Inorganic ion transport and metabolism* | |
| EEB69373 | Putative heme degradation protein | 4.5 |
| EEB69747 | Extracellular solute-binding protein, family 1 | 5 |
| | *S: Function unknown* | |
| EEB69472 | Phasin family protein | -8.1 |
| | *T: Signal transduction mechanisms* | |
| EEB70997 | Serine protein kinase, PrkA | -6.9 |

Table S5: Proteins differentially expressed in Δ*varR* biofilm cells (ΔVB) compared to WT biofilm cells (WTB). The average fold change in expression in ΔVB is shown, with grey shading indicating down-regulation. Proteins were detected with $p < 0.05$ across two biological replicates except where * denotes that were proteins identified in three biological replicates. Shading denotes proteins that were down regulated in expression in ΔVB relative to WTB. Genbank accession numbers are given along with the COG category and protein description provided in the NCBI database. The qualifier ^ denotes that a protein with the same accession number was differentially regulated in the WT biofilm proteome (Table S3). Underlined accession numbers indicate that the protein was also differentially expressed in Δ*varR* relative to WT under planktonic growth conditions (Table S4)

| Accession number | NCBI Protein Annotation | Fold change in expression |
|---|---|---|
| | *C: Energy production and conversion* | |
| EEB69852^ | Aconitate hydratase | 4* |
| EEB70986^ | Pyruvate carboxylase | -2 |
| EEB72814 | Citrate synthase I | -6.1* |
| | *E: Amino acid transport and metabolism* | |
| EEB72300 | ABC-type amino acid transporter, periplasmic binding | 2.5 |
| EEB69952^ | Arginase | -1.4 |
| EEB72013^ | ABC-type peptide transporter, substrate-binding | -1.4 |
| EEB70210 | Glutamate dehydrogenase 1 | -1.9 |
| EEB70952 | ABC-type spermidine/putrescine transporter, ATPase | -2.6 |
| | *I: Lipid transport and metabolism* | |
| EEB70651^ | Acetoacetyl-CoA reductase | -3.1 |
| EEB71680^ | Acetyl-CoA acetyltransferase | -8.2 |
| EEB71107^ | Polyhydroxyalkanoate depolymerase, intracellular | -8.8 |
| | *J: Translation, ribosomal structure and biogenesis* | |
| EEB72106^ | Ribosomal protein S1 | 4.5 |
| EEB71543^ | Ribosomal protein L15 | 4.8* |
| EEB69437^ | Ribosomal protein L3 | 5.1 |
| | *M: Cell wall/membrane/envelope biogenesis* | |
| EEB70816^ | Outer membrane protein assembly complex, YaeT protein | 3.8 |
| | *M: Cell wall/membrane/envelope biogenesis/ U: Intracellular trafficking and secretion* | |
| EEB72298 | Outer membrane efflux protein, TolC family | 1.7 |
| | *N: Cell motility/ T: Signal transduction mechanisms* | |
| EEB69630^ | Methyl-accepting chemotaxis sensory transducer | 6.1 |
| | *O: Posttranslational modification, protein turnover, chaperones* | |
| EEB70734 | Bacterial trigger factor protein | 2.9 |
| | *Q: Secondary metabolites biosynthesis, transport and catabolism* | |

| | | |
|---|---|---|
| EEB71180 | Extracellular solute-binding protein, family 7 | 7.2 |
| | *R: General function prediction only* | |
| EEB69264^ | Predicted epimerase, PhzC/PhzF homolog | -2 |
| EEB69320^ | Alpha/beta hydrolase family | -3.4 |
| | *S: Function unknown* | |
| EEB70321^ | Predicted periplasmic protein | -2.7 |
| EEB69472^ | Phasin family protein | -17* |
| | *T: Signal transduction mechanisms* | |
| EEB70997^ | Serine protein kinase, PrkA | -2.3* |

**References**

Ausubel, F., Brent, R., Kingston, R., Moore, D., Siedman, J., and Smith, J. (1999). *Short Protocols in Molecular Biology*. New York, NY: John Wiley & Sons, Inc.

Katzen, F., Becker, A., Ielmini, M. V., Oddo, C. G., and Lelpi, L. (1999). New mobilizable vectors suitable for gene replacement in Gram negative bacteria and their use in mapping of the 39 end of the Xanthomonas campestris pv. campestris gum operon. *Appl. Environ. Microbiol.* 65, 278–282.

Kovach, M. E., Phillips, R.W., Elzer, P. H., Roop, R.M., and Peterson, K.M. (1994). pBBR 1 MCS: a broad host range cloning vector. *Biotechniques* 16, 800–802.
